# Covalent Grafting of Cationic Polythiophene Nanowires onto a Polyurethane Interface via Surface-Assisted Polymerization

**DOI:** 10.1021/acs.langmuir.6c00484

**Published:** 2026-06-12

**Authors:** Sezer Özenler, Muge Yucel, Ümit Hakan Yıldız

**Affiliations:** † Department of Chemistry, 52972 Izmir Institute of Technology, Urla, Izmir 35430, Turkey; § Department of Bioengineering, Izmir Institute of Technology, Urla, Izmir 35430, Turkey; ∥ Department of Photonics, Izmir Institute of Technology, Urla, Izmir 35430, Turkey; ⊥ Polymer Science and Engineering Program, Izmir Institute of Technology, Urla, Izmir 35430, Turkey

## Abstract

We report a robust and scalable approach for covalently grafting cationic polythiophene (CPT) nanowires onto polyurethane (PU)-functionalized gold surfaces using a surface-assisted (SurfAst) polymerization strategy. The methodology involves sequential incubations of hexamethylene diisocyanate (HDI) and 1,4-butanediol (1,4-BDO) on an 11-mercaptoundecanoic acid (MUA)-functionalized gold substrate, forming an OH-terminated PU interface. Subsequent functionalization with 2-isocyanatoethyl methacrylate yields a methacrylate-terminated PU interface, enabling the grafting of CPT nanowires through an UV-induced reaction between the methacrylate group on the PU interface and the allyl moieties of CPT. Atomic force microscopy (AFM) reveals the formation of highly elongated and well-defined CPT nanowires with stability. Electrostatic force microscopy (EFM) further confirms distinct phase shifts in the CPT-grafted regions. These findings demonstrate not only the successful immobilization of CPT on the PU-functionalized gold surface but also the preservation of accessible cationic functionalities, which ensure surface activity. A key aspect of this platform is the use of ethylene glycol (EG) as the solvent for CPT during the grafting process, which facilitates well-dissolved CPT chains and may provide the polymer in a dispersed state in solution, allowing effective interaction with the functionalized surface. These results establish a chemically robust and electrostatically tunable CPT–PU interphase, offering new opportunities for integration into next-generation electronic and sensing technologies.

## Graphical abstract

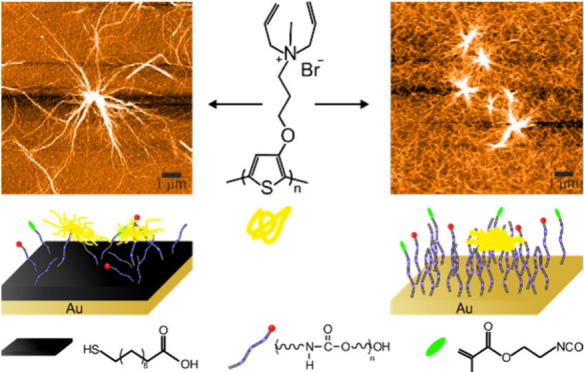

## Introduction

Conjugated polymers (CPs) represent a versatile class of materials with highly tunable optical and charge-transport properties. Their aromatic backbones enable controlled electron delocalization, directly affecting photophysical behavior and electronic performance, which makes CPs highly attractive for bioelectronics and sensing applications.^1,2^ In such systems, the electrical response of CPs is strongly influenced by their molecular structure and conformation, which could span a broad range of morphological order from fully amorphous to polycrystalline. Therefore, the structural and chemical characteristics of conjugated polymer interfaces play a decisive role in determining device performance.^3,4^

In device fabrication, CPs are commonly deposited onto substrates using techniques such as dip-coating, spin-coating, or roll-to-roll processing, where attachment typically relies on secondary interactions. However, regarding the long-term performance, these noncovalent coatings are susceptible to ambient conditions, steam exposure, and solvent contact, which may induce delamination of the film and impaired charge-transfer characteristics.^5,6^ To address these limitations, covalent “grafting to” strategies have been developed to immobilize conjugated polymers on surfaces, thereby improving film robustness, mechanical stability, and solvent resistance.^7,8^ By effectively “freezing” polymer chains in defined positions, covalent grafting has been shown to enhance charge transport and overall electronic performance.^9–11^ Although several surface-grafting approaches exist, only a limited number of studies have demonstrated the covalent immobilization of CP derivatives. For example, Martin et al. reported thiol–ene click chemistry to graft functionalized poly­(thiophene)­s onto (3-mercaptopropyl)­triethoxysilane-modified SiO_2_ wafers, yielding well-oriented, electroactive, and solvent-resistant ultrathin films.^12^ In addition, Davis et al. further investigated the effect of photoinduced the use of thiol–ene surface grafting on the optoelectronic properties of end-group and side-chain functionalized conjugated poly­(fluorene)^11^ while Youm et al. employed surface-initiated Kumada catalyst-transfer polymerization to prepare surface-grafted polythiophene thin films.^13^ These studies highlight the feasibility of immobilizing polythiophene through covalent grafting approaches while controlling film structure and thickness.

For ionic pendant group-bearing conjugated polythiophenes (CPTs), which offer extensive opportunities for versatile chemical modification, achieving high grafting densities remains challenging due to the need for precise control over polyelectrolyte conformation.^9,14^ Chen et al. synthesized a regioregular poly­(3-hexylthiophene)-*block*-poly­(3-bromohexylthiophene) (P3HT-*b*-P3BrHT) block copolymer and reported nanowire formation driven by π–π stacking in P3HT-*b*-P3BrHT with 70:30 and 50:50 block ratios. However, P3BrHT content exceeding 50% significantly disrupted chain regularity and reduced crystallization.^15^ Similarly, Danielsen et al. synthesized P3BrHT/P3HT copolymers with varying charge ratios by the Menshutkin amine quaternization reaction and found persistence lengths of approximately 3 nm across all charge ratios, suggesting negligible electrostatic rigidity for conjugated polyelectrolytes with pendant charges in dilute solutions.^2^ Both theoretical and experimental studies have emphasized the significant role of solvent interactions in tuning polymer conformation, where strong polymer–solvent interactions over polymer–polymer interactions promote an extended polymer chain, increase grafting density, and concurrently preserve the photophysical properties.^16–18^

Building on these advancements, we previously introduced nanometer-precise polyurethane (PU) interfaces, electroactive nanogels, and thickness-gradient polymer coatings through a surface-assisted (SurfAst) polymerization method.^19–22^ This methodology, based on the interaction between gold and isocyanate groups, enables sequential incubations of alkanediol and diisocyanate monomers under mild conditions, without requiring inert atmospheres, degassing, or costly catalysts. Here, we present a straightforward and modular methodology for grafting poly­(*N*-allyl-*N*-methyl-*N*-(3-((4-methylthiophen-3-yl)­oxy)­propyl)­prop-2-en-1-aminium bromide), a cationic conjugated polythiophene (CPT), onto 11-mercaptoundecanoic acid (MUA)-functionalized gold surfaces via SurfAst urethane polymerization. Sequential incubations of hexamethylene diisocyanate (HDI) and 1,4-butanediol (1,4-BDO) produced an OH-terminated PU interface. Subsequent incubation with 2-isocyanatoethyl methacrylate generated a methacrylate-terminated PU layer that enables UV-induced covalent grafting of allyl groups in the CPT. This study presents an immobilization protocol for CPT in ethylene glycol (EG) medium, in which the EG environment mitigates challenges associated with ionic polythiophene grafting by reducing polymer–polymer interactions and enhancing grafting efficiency (Scheme 1, left).

**Scheme 1:**
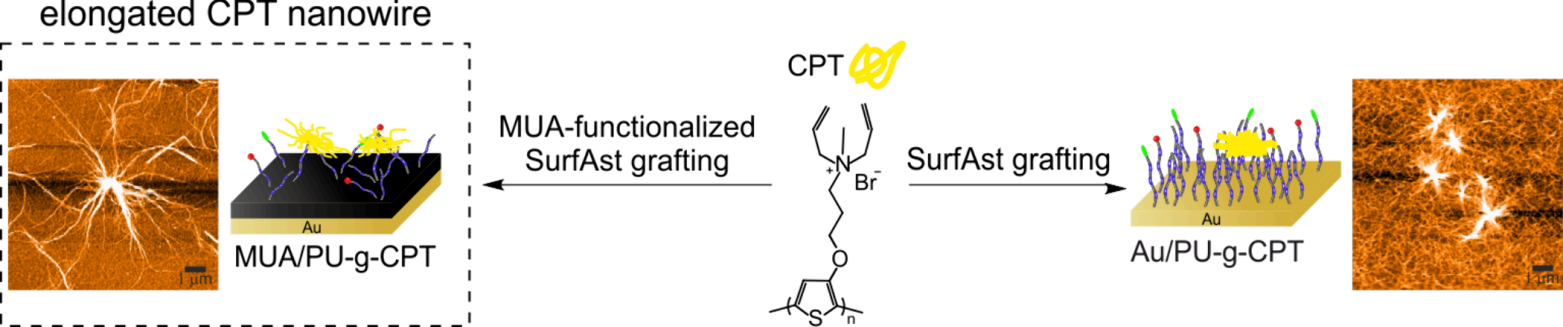
Illustration of the Fabricated CPT Nanowires on Gold Surfaces (MUA/PU-g-CPT and Au/PU-g-CPT)

## Experimental Section

All chemicals were purchased from Sigma-Aldrich and used without further purification.

### Steps Included in Surface Functionalization

#### MUA Functionalization of Gold Surface

A gold surface is incubated in 1 mM MUA EtOH solution for 1 day. The surface is thoroughly washed with EtOH following incubation.

#### PU Formation

HDI (70 μL in 5 mL of acetone, 200 μL of DBTDL catalyst) is incubated in acetone at 40 °C for 20 min, then the surface is washed with acetone. Subsequently, 1,4-BDO (40 μL in 5 mL of acetone, 200 μL of DBTDL catalyst) is incubated on the gold surface at 40 °C for 20 min, the surface is washed with acetone. These two incubation processes are repeated six times to obtain an OH-terminated PU interface.

#### Methacrylate Termination

The OH-terminated PU interface is incubated with 2-isocyanate ethyl methacrylate (50 μL in 5 mL of acetone, 200 μL of DBTDL catalyst) at 40 °C for 20 min and rinsed with plenty of acetone to yield methacrylate-terminated PU interface.

#### CPT Photografting

100 μL of ethylene glycol (EG) solution of 2.1 mg/mL poly­(*N*-allyl-*N*-methyl-*N*-(3-((4-methylthiophen-3-yl)­oxy)­propyl)­prop-2-en-1-aminium bromide (CPT) with 1.8% Irgacure is dropped over the surface (methacrylate-terminated PU interface) which is subsequently irradiated for 7 min (λ = 365 nm). Large amounts of pure water (an approximate total volume of 500 mL) are used to rinse the gold surface.

#### EG/Irgacure Treatment

1.8% Irgacure solution (EG) is dropped over the surface (methacrylate-terminated PU interface) which is subsequently irradiated for 7 min (λ = 365 nm). The surface was then rinsed with pure water.

All surfaces were prepared independently of each other (Table 1).

**Table 1:**
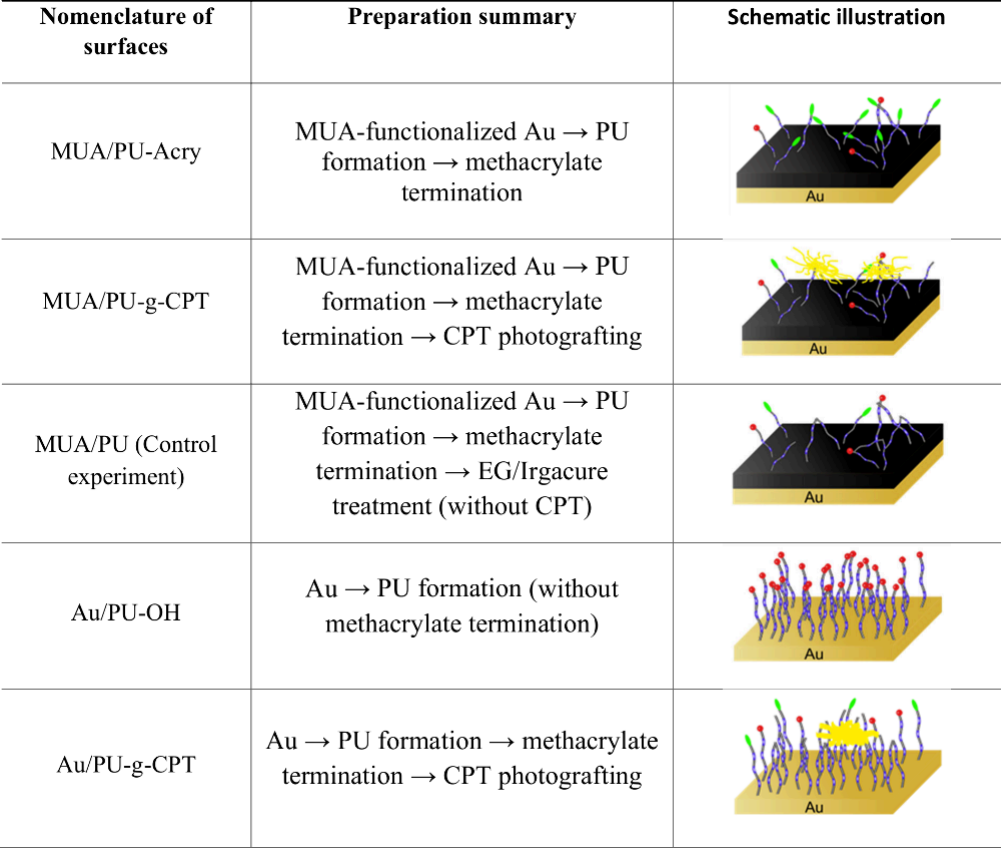
Summary of the Surfaces Used in This Study

Nanosurf AFM (Stat0.2LAuD, static force) was used for the topographical characterization of gold surfaces. To investigate electrostatic properties, electrostatic force microscopy (EFM) measurements were performed by double-pass mode with a 40 nm height, and the surface was charged by applying 0, +2, and −2 V at room temperature. To calculate phase shift values for each sample, cross-sectional analysis was performed on the EFM phase images by extracting line profiles across the full scan width (10 μm). The phase shift (|Δφ|) was determined from the resulting phase-distance profiles as the absolute difference between the peak phase signal and the average background signal, calculated from regions without discernible surface features. Multiple profiles from different areas of each sample were analyzed to obtain mean values and standard deviations. AFM and EFM images were processed using the Nanosurf analysis software with line-fit correction applied to minimize background slope and scanning artifacts prior to analysis. Infrared measurements were performed using a PerkinElmer Spectrum 100 FTIR equipped with VeeMAX III variable angle specular reflectance accessory. XPS mapping analyses were performed over a 1 × 1 mm area using a monochromatic Al Kα source (1486.68 eV) at a pass energy of 30 eV and a spot size of 30 μm (3 scans). XPS point analyses were acquired with a 400 μm spot size. The resulting XPS mappings were constructed from multiple point spectra and processed by PCA-based multivariate analysis, allowing the dominant spectral component in each region to be visualized.

## Results and Discussion

### Surfaces in Fabrication Steps of CPT Nanowires

The sequential fabrication of CPT nanowires is illustrated in Scheme 2. Initially, the gold surface was functionalized with 11-mercaptoundecanoic acid (MUA) to introduce carboxylic acid (−COOH) groups. The well-ordered MUA-functionalized surface was then incubated with hexamethylene diisocyanate (HDI). During this step, a potential reaction occurs between the isocyanate groups of HDI and the carboxylic acid groups of MUA, which are loosely organized on the gold surface.^23^ To validate this reaction, a reference surface (stamped-Au/MUA/PU-OH) was prepared, and a detailed preparation protocol and AFM topography image are given in the Supporting Information. (Scheme S1 and Figure S1). Following HDI incubation, an NCO-terminated interface was successfully generated on the MUA-functionalized surface. Subsequent sequential reactions with 1,4-butanediol (1,4-BDO) and HDI established an OH-terminated polyurethane (PU) interface on the gold surface. The OH-terminated PU interface, derived from the weakly organized MUA-functionalized domains, was further incubated with 2-isocyanate ethyl methacrylate to yield a methacrylate-terminated PU interface, designated as MUA/PU-Acry, which was subsequently utilized in the fabrication of MUA/PU-g-CPT and MUA/PU.

**Scheme 2:**
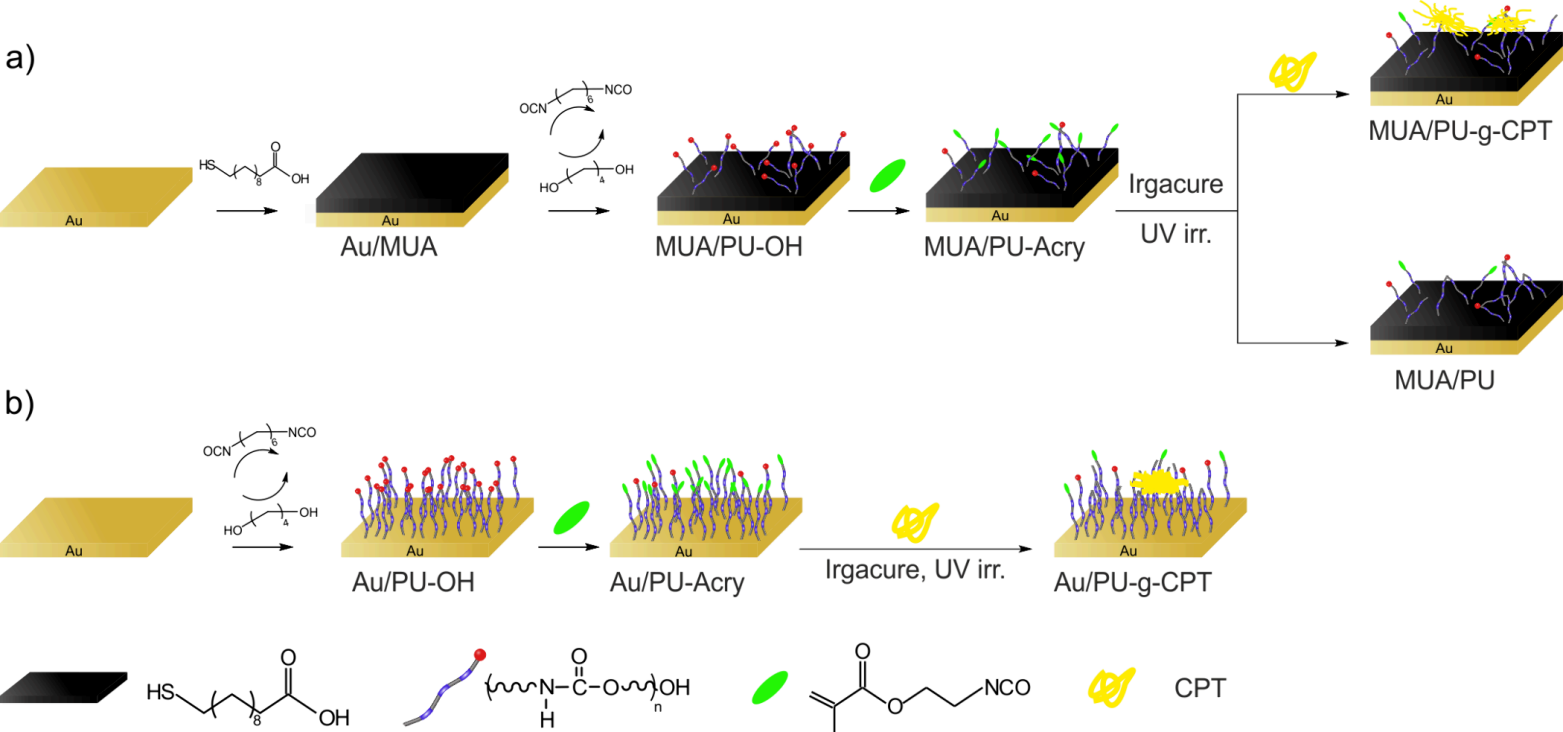
Schematic Illustration of the Surfaces: (a) 11-Mercaptoundecanoic Acid (MUA) Base and (b) Bare Gold Base

MUA/PU-g-CPT was fabricated by UV irradiation of MUA/PU-Acry in ethylene glycol (EG) solution containing allyl group-bearing conjugated polythiophene (CPT) and Irgacure as a photoinitiator. Under UV irradiation, methacrylate groups on MUA/PU-Acry undergo cross-linking with allyl groups of CPT, which contains multiple attachment points, resulting in covalently grafted CPT nanowires on the PU interface.^24^ Direct adsorption/grafting of conjugated polythiophenes on metallic substrates can substantially influence chain conformation and electronic structure, leading to modified optical and redox behavior. Introducing a nanometer-scale PU interlayer provides both a reactive platform for controlled photografting and a spacer that reduces direct polymer–metal interactions. Consequently, the PU interlayer may serve three complementary functions: increasing local methacrylate density for efficient grafting, partially decoupling CPT electronically from the gold substrate, and tuning CPT–solvent interactions at the interface.

As a control, MUA/PU was prepared by irradiating MUA/PU-Acry under identical conditions without the addition of CPT. To further confirm the methodology, the same procedure used for the fabrication of MUA/PU-g-CPT was applied for Au/PU-g-CPT, where CPTs are cross-linked to a SurfAst modified-bare gold substrate without MUA functionalization.

To assess the coating efficiency of HDI/1,4-BDO cycles during SurfAst polymerization, gold substrates were analyzed by high-resolution X-ray photoelectron spectroscopy (XPS) chemical mapping for C 1s, N 1s, O 1s, and Au 4f after 4, 6, and 8 reaction cycles. One 2-cycle sequence corresponds to HDI incubation followed by 1,4-BDO, which is expected to yield urethane on the surface. XPS mapping results reveal the spatial distributions and abundance of carbon, nitrogen, and oxygen across the gold substrates (Figures S2–S4). Quantitatively, the measured Au 4f signal intensities are 0.971 (4 cycles), 0.575 (6 cycles), and 0.901 (8 cycles). The reduced Au 4f signal after six cycles indicates a more continuous PU coating since a lower Au signal reflects fewer exposed substrate regions within the analysis depth. In contrast, an increased Au signal at eight cycles suggests partial re-exposure of the gold surface. A possible mechanism for the increased Au signal beyond six cycles is the reorganization of adjacent urethane groups, which could adopt a more perpendicular orientation due to hydrogen bonding interactions, despite the additional monomer deposition at eight cycles. In addition to monitoring the Au 4f abundance, coating uniformity was evaluated through binary and tertiary chemical mapping (Figures S3 and S4), as well as quaternary mapping shown in Figure 1. The XPS quaternary chemical mapping for Au–C–N–O exhibits strong correlation with the elemental mapping trends. When evaluating the XPS mapping of Au in relation to the efficiency of the PU coating, the quantified Au abundance values were recorded as 5.5 ± 0.11% after four cycles, 1.9 ± 0.06% after six cycles, and 8.9 ± 0.14% after eight cycles. In addition, AFM images in our earlier study show the emergence of rodlike domains together with nanoporous structures at the PU interface after eight cycles, which may lead to local thinning or increased heterogeneity of the coating, thereby contributing to the increased apparent Au signal at higher cycle numbers.^19^ These observations indicate that the application of six cycles yields the most effective and uniform PU interface, as reflected by minimal Au exposure. Figure 1d–g shows a systematic decrease in the Au 4f signal and an increase in the PU-related C 1s, N 1s, and O 1s signals with increasing cycle number. In other words, increasing the number of SurfAst cycles progressively shifts the XPS count values from Au-dominated toward C/N/O-dominated, consistent with growing organic surface coverage.

**Figure 1:**
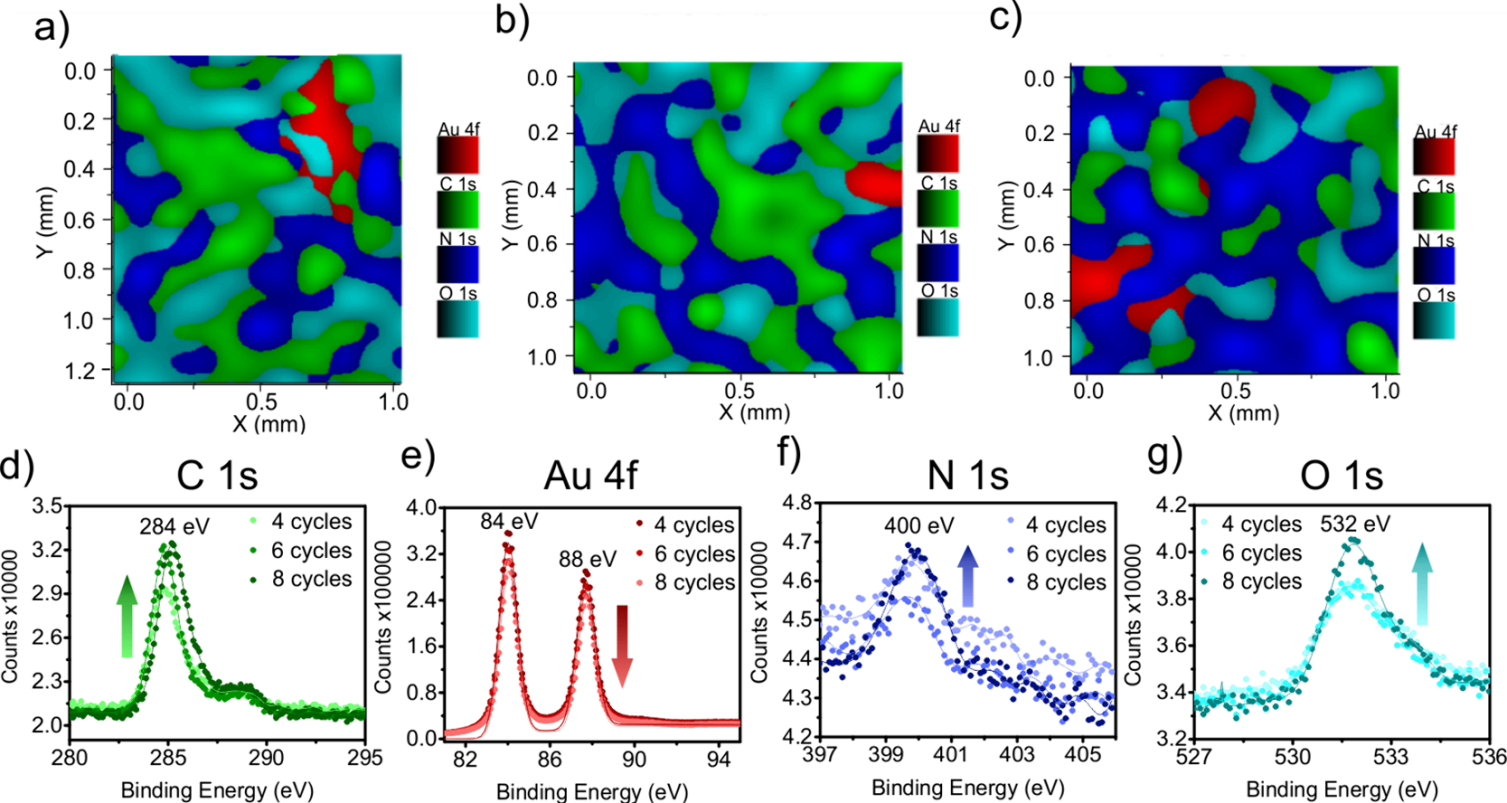
XPS quaternary chemical mapping of the bare gold surface after (a) 4, (b) 6, and (c) 8 cycles of HDI/1,4-BDO incubation. Color coding: green, C 1s; blue, N 1s; cyan, O 1s; and red, Au 4f. (d–g) Corresponding high-resolution XPS spectra for the C 1s, Au 4f, N 1s, and O 1s regions after 4, 6, and 8 cycles.

### AFM Topography Analysis of the Surfaces

Atomic force microscopy (AFM) topography analysis was employed to characterize the surface morphology of each surface. Multiple cross-sectional and surface area profiles were collected to extract key morphological parameters, including feature height, width, and length, as well as roughness. Cross-sectional profiles were taken along the dashed cyan lines indicated in the corresponding topography AFM images and are provided in Figure S5.

AFM images along with schematic illustrations of the surfaces are presented in Figure 2, and the morphological parameters of corresponding surfaces are listed in Table 2. Figure 2a displays the methacrylate-terminated PU interface on the gold substrate (MUA/PU-Acry), which forms thread-like PU fibers. The PU fibers on MUA/PU-Acry exhibit an area roughness of 50 (±15) nm, whereas the well-ordered MUA regions show an area roughness of 4.4 (±0.9) nm, which is slightly higher than the roughness of bare gold (2.4 nm). However, localized sphere-like structures on the MUA domains increase the roughness up to ∼15 nm. The PU fibers display well-defined features, with centers averaging 38 (±8) nm in height, and fibers with an average height of 10 (±2) nm, a width of 159 (±35) nm, and a length of 2.0 (±0.5) μm.

**Figure 2:**
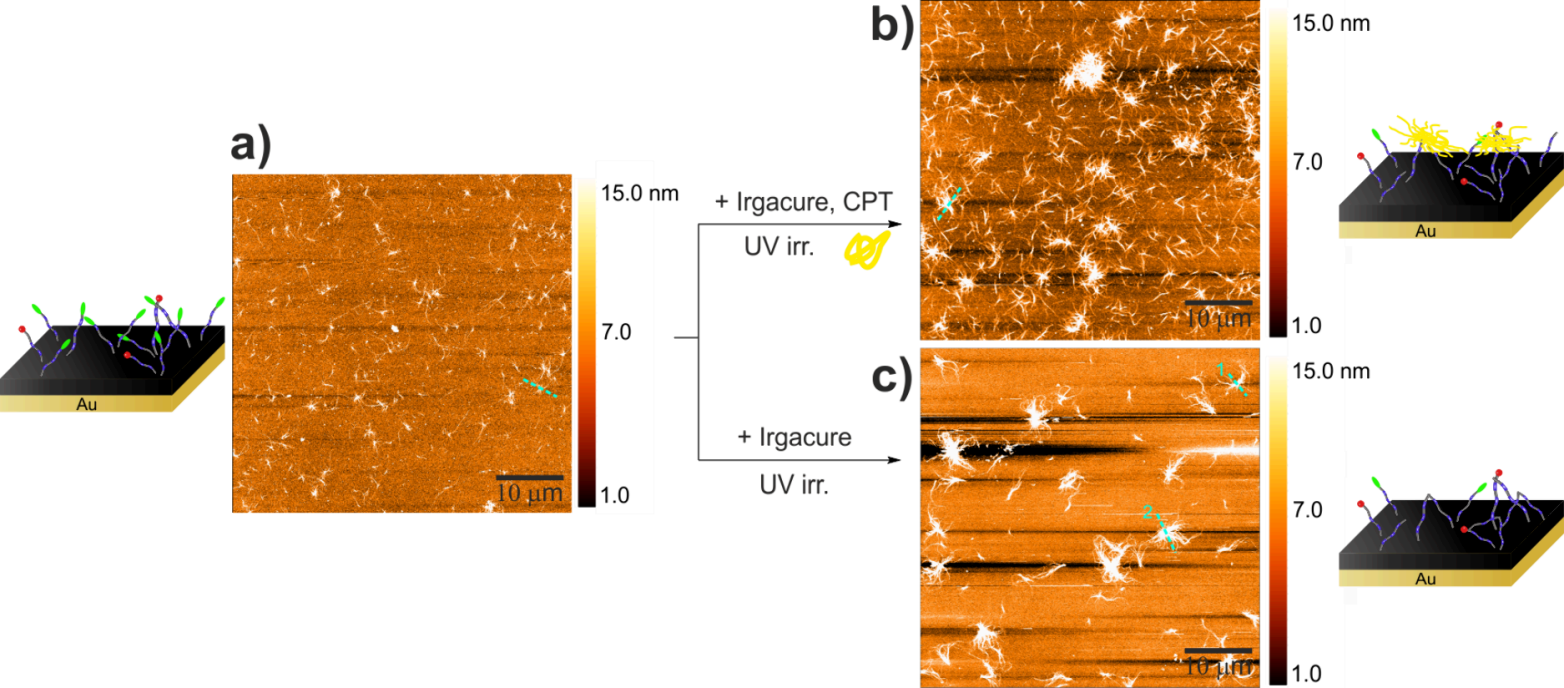
AFM topography images of (a) MUA/PU-Acry, (b) MUA/PU-g-CPT, and (c) MUA/PU with corresponding schematic illustrations.

**Table 2:** Quantitative Summary of the Morphological Parameters Extracted from AFM Cross-Section and Surface Area Analyses of the Surfaces in Figure 2

	roughness (nm)		height center (nm)	height fiber (nm)	width fiber (nm)	length fiber (μm)
MUA/PU-Acry	50 (±15)		38 (±8)	10 (±2)	159 (±35)	2.0 (±0.5)
MUA/PU-g-CPT	93 (±35)		61 (±29)	19 (±5)	265 (±110)	3.6 (±0.7)
MUA/PU	138 (±70)	(i)	53 (±14)	10 (±3)	166 (±63)	1.6 (±0.2)
		(ii)	220 (±36)	11 (±2)	189 (±100)	2.5 (±0.5)

MUA/PU-Acry was subsequently exposed to UV irradiation in EG solution containing CPT and Irgacure to obtain MUA/PU-g-CPT through cross-linking between methacrylate groups on the PU interface and allyl groups of CPT. The AFM image of the CPT-grafted surface in Figure 2b reveals elongated nanowire-like structures. To evaluate the morphology of CPT chains, ethylene glycol solution of CPT drop-cast onto gold substrate. In contrast to the functionalized surface, the drop-cast CPT exhibits a globular morphology, suggesting that the elongated structures arise from the organization of CPT chains after covalent grafting onto PU interface (Figure S6). The resulting CPT nanowires exhibit an area roughness of 93 (±35) nm, while the well-ordered MUA regions retain a similar roughness to MUA/PU-Acry (3.4 (±0.9) nm). CPT nanowires form densely packed centers with an average height of 61 (±29) nm. The fibers extending from these centers have an average height of 19 (±5) nm and a width of 265 (±110) nm, tapering to heights of 6 (±2) nm and widths of 58 (±40) nm at their distal ends, with an overall length of 3.6 (±0.7) μm. These structures maintain stability for approximately a year after fabrication.

To confirm the reactivity of the methacrylate groups on MUA/PU-Acry, a control surface (MUA/PU) was prepared by UV irradiation of MUA/PU-Acry in an EG solution containing Irgacure without CPT. As shown in Figure 2c, the photoinitiator triggers cross-linking exclusively between neighboring methacrylate groups, generating interwoven PU structures across the surface. These PU networks exhibit an average area roughness of 138 (±70) nm, while the MUA region maintains a roughness of 4.7 (±0.7) nm, consistent with the surfaces, MUA/PU-Acry and MUA/PU-g-CPT. Two distinct morphological populations are observed on MUA/PU: (i) a less aggregated form with average central heights of 53 (±14) nm and fibers of 10 (±3) nm in height, 166 (±63) nm in width, and 1.6 (±0.2) μm in length; and (ii) a more prominent aggregated population with average central heights of 220 (±36) nm and fibers of 11 (±2) nm in height, 189 (±100) nm in width, and 2.5 (±0.5) μm in length.

Compared to MUA/PU-g-CPT, MUA/PU exhibits more heterogeneous and randomly interconnected networks with increased roughness. The smaller features (i) on MUA/PU show cross-section profiles similar to those of MUA/PU-Acry; however, the larger aggregates (ii) deviate substantially from both MUA/PU-Acry and MUA/PU-g-CPT, particularly in the height of central domains. Without a CPT partner, the photoinitiated methacrylate–methacrylate coupling proceeds in an uncontrolled manner, producing larger and irregular aggregates that differ from the organized nanowire architecture observed in MUA/PU-g-CPT. On the contrary, the CPT nanowires on MUA/PU-g-CPT display a more defined hierarchy between center and fiber height with significantly longer fiber extensions. The topographical features on MUA/PU-g-CPT reflect the combined contribution of the underlying surface functionalization^25^ and the solvent-mediated conformational control of the CPT chain in ethylene glycol (EG).^26^ As we previously discussed, the good solvent effects of EG on cationic CPT increase the excluded volume of the CPT chain through polymer–solvent interactions driven in part by its high hydrogen-bonding component (δH).^27–29^ This suppresses polymer–polymer aggregation and promotes chain elongation, yielding defined and thin nanowire structures with high grafting density.^30^ To further verify the solvent-driven contribution, small-angle neutron scattering (SANS) measurements of CPT in EG and water revealed lower power-law exponents in EG, supporting enhanced CPT–solvent interactions and reduced interchain association in the EG medium (Figure S7).

Au/PU-g-CPT was prepared to further validate the surface reactions in the absence of MUA functionalization, thereby distinguishing its characteristics from those of MUA/PU-Acry, MUA/PU-g-CPT, and MUA/PU. As shown in Figure 3a, the bare gold substrate exhibits a smooth morphology with an average area roughness of 2.4 (±0.27) nm. The OH-terminated PU interface was formed on the bare gold using the SurfAst urethane polymerization method based on isocyanate-gold interaction.^19,31^ After 6-cycle SurfAst urethane polymerization onto bare gold, designated as Au/PU-OH, the average area roughness was found to be ∼50 nm (Figure 3b). Subsequent UV-induced immobilization of CPT in an EG solution containing Irgacure yielded Au/PU-g-CPT, as presented in Figure 3c. The CPT-grafted regions exhibit an average area roughness of 123 (±43) nm, whereas the nongrafted PU regions display a roughness of 23 (±4) nm, significantly greater than the domains observed in MUA/PU-Acry, MUA/PU-g-CPT, and MUA/PU. The AFM topography image reveals the formation of numerous short, filament-like CPT structures growing outward from a central region. These features possess an average central height of 125 (±33) nm and filament-like extensions characterized by an average height of 64 (±20) nm, a width of 290 (±33) nm, and a length of 0.6 (±0.1) μm; a quantitative summary of these morphological parameters is provided in Table 3. Compared to nanowires on MUA/PU-g-CPT, the substantially greater heights of the CPT structures on Au/PU-g-CPT are consistent with findings from the study investigating the thickness of an amine-functionalized conjugated polymer during deposition.^25^ These observations confirm that the PU interface can be effectively postfunctionalized by the reaction between the methacrylate-terminated PU and allyl-functionalized CPT. The S 2p spectrum (Figure S8) exhibits a characteristic doublet with the S 2p_3/2_ peak centered at ∼164.0 eV and the S 2p1/2 peak at ∼165.2 eV (splitting of 1.18 eV, area ratio 2:1). These peaks correspond to the neutral sulfur atoms within the thiophene rings of the polythiophene backbone. A secondary, broader doublet at higher binding energy (167–169 eV) is attributed to highly oxidized sulfur species (SO_*x*_), such as sulfonates or sulfates. These species likely originate from the oxidation of sulfur of thiophenes by air or dissolved oxygen in incubation solutions. The stability of CPT nanowires on the surface was tested by exposing samples to ambient conditions for 2 weeks XPS survey spectra of aged samples are presented in Figure S9, confirming the presence of CPT on the surface. Both aged Au/PU-g-CPT and MUA/PU-g-CPT samples exhibited S 2p signals at 163 and 168 eV, which arise from the grafted polythiophene on the surface. The binding energy at 163 eV is attributed to the S 2p of thiophene sulfur atoms (C–S–C); on the other hand, the signal at 168 eV is assigned to SOx species generated by the partial oxidation of sulfur by atmospheric O_2_. This higher S 2p signal intensity is ascribed to the preservation of CPT even after incubation under ambient conditions. A physiosorbed layer would likely be removed or exhibit a neutral phase response similar to the controls. Overall, these results demonstrate the robust stability of grafted polythiophene under aging conditions.

**Figure 3:**
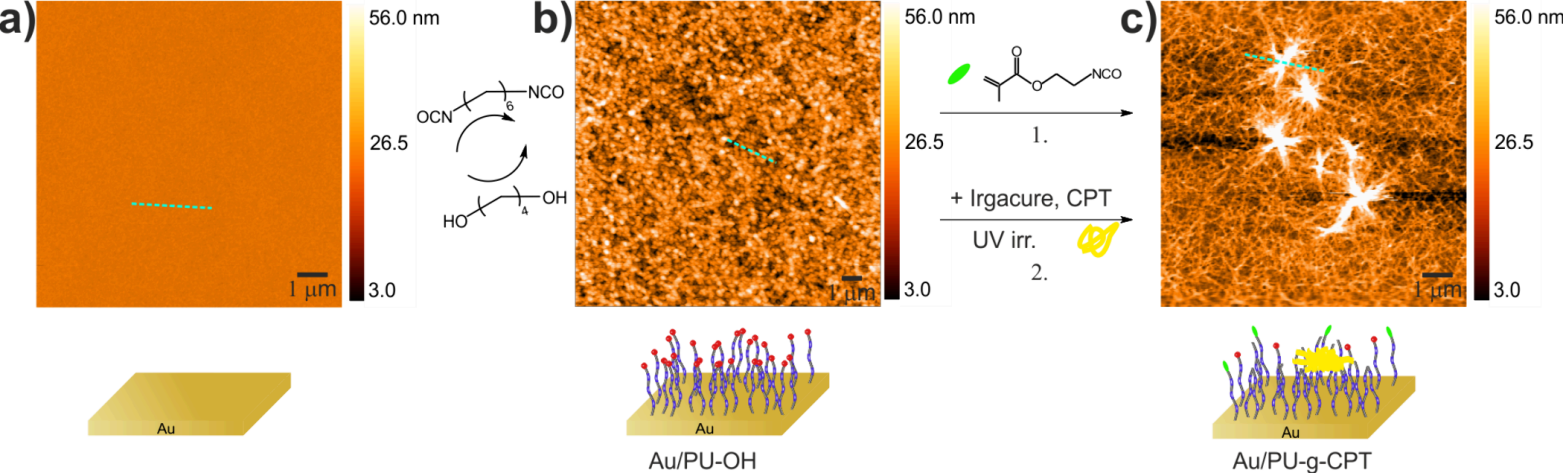
AFM topography images of (a) gold, (b) Au/PU-OH and (c) Au/PU-g-CPT with corresponding schematic illustrations.

**Table 3:** Quantitative Summary of the Morphological Parameters Extracted from AFM Cross-Section and Surface Area Analyses of the Surfaces in Figure 3

	roughness (nm)	height center (nm)	height fiber (nm)	width fiber (nm)	length fiber (μm)
Au surface	2.4 (±0.27)				
Au/PU-OH	∼50				
Au/PU-g-CPT	123 (±43)	125 (±33)	64 (±20)	290 (±33)	0.6 (±0.1)

### Electrostatic Force Microscopy (EFM) Analysis of the Surfaces

EFM was employed to probe the electrostatic interactions between the AFM tip and surface structures under constant applied voltage and tip lift. In these measurements, the tip voltage (*V*_tip_) was set to ±2 V to maximize the phase contrast between the polymeric domains and the gold surface (PU domain for Au/PU-g-CPT). The phase-tip voltage graphs for MUA/PU-Acry and MUA/PU are given in Figure S10. A *V*_tip_ of 0 V was recorded for each surface as a reference measurement. All measurements were conducted in double-pass mode with a 40 nm second tip lift. Figure 4 presents the EFM images and the corresponding phase shift profiles for the surfaces, MUA/PU-Acry, MUA/PU-g-CPT, MUA/PU, and Au/PU-g-CPT under tip voltages of ±2 V, along with the 0 V profiles for comparison. Phase shift profiles, represented below the corresponding micrograph, were extracted along the cyan lines to encompass the center, fiber, and substrate.

**Figure 4:**
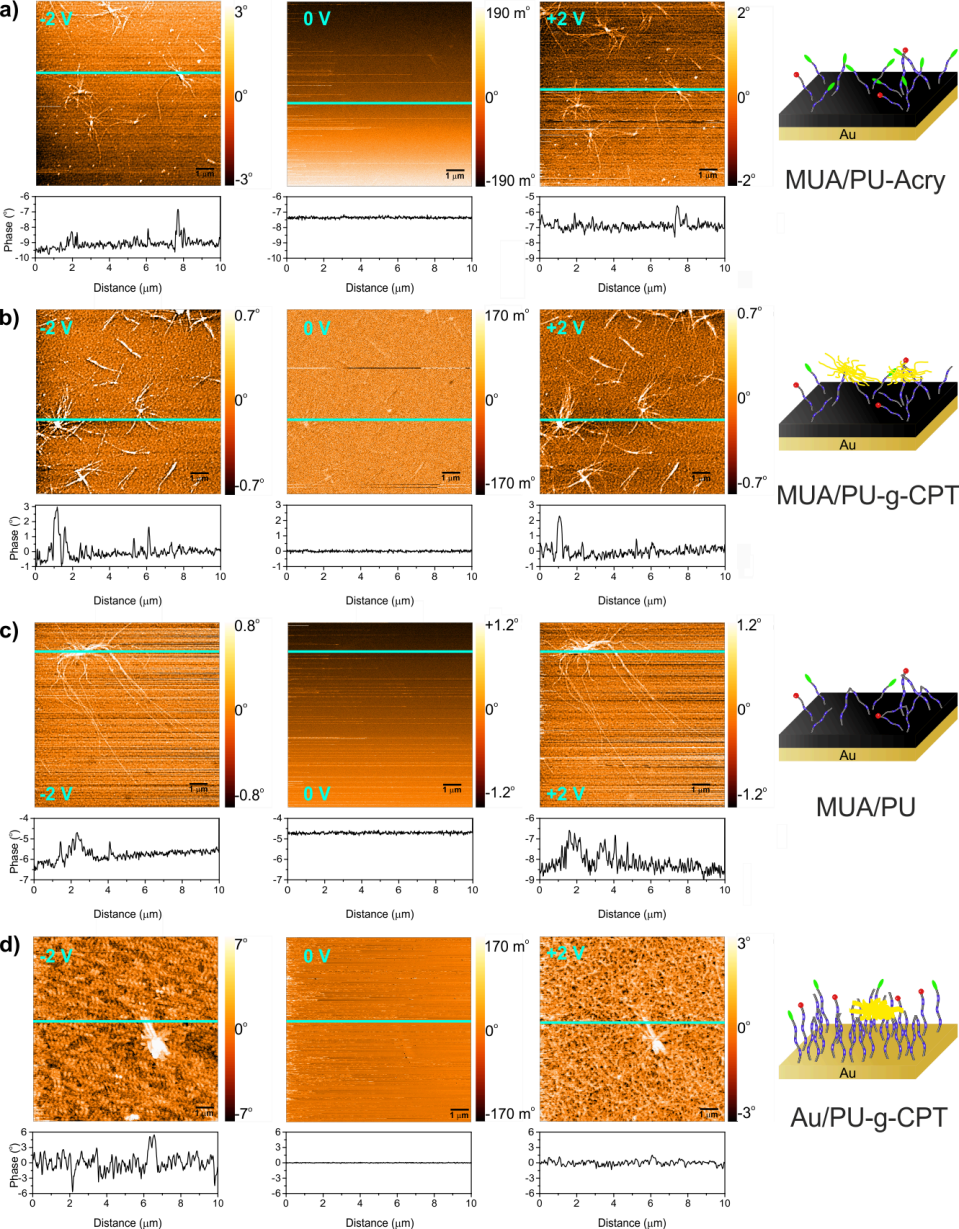
EFM images and phase-shift profiles of (a) MUA/PU-Acry, (b) MUA/PU-g-CPT, (c) MUA/PU, and (d) Au/PU-g-CPT at *V*_tip_ = ±2 and 0 V as a reference measurement.

For MUA/PU-Acry, the phase shifts are found to be approximately 2.3° (±0.2°) at *V*_tip_ = −2 V and 1.4° (±0.2°) at +2 V (Figure 4a), indicating slightly stronger interactions with the negatively charged tip. In MUA/PU-g-CPT, where cationic CPT nanowires are present, EFM measurements in Figure 4b revealed a phase shift of 3.0° (±0.3°) and 2.3° (±0.3°) phase shifts at *V*_tip_ = −2 and +2 V, respectively. The resulting 0.7° phase shift difference demonstrates the expected stronger electrostatic interaction induced by the negatively charged tip. In contrast, the control surface (MUA/PU) shows nearly identical phase shifts of 1.0° (±0.2°) at −2 V and 1.8° (±0.2°) at +2 V, consistent with the absence of CPT and the predominant PU-based composition. These results closely match those of MUA/PU-Acry, supporting the similarity in their surface chemistry. The other control surface (Au/PU-g-CPT) is demonstrated in Figure 4d to provide further insight into CPT immobilization on the PU-functionalized gold surface under constant analysis parameters. Phase shifts of 5.6° (±0.4°) at *V*_tip_ = −2 V and 1.6° (±0.2°) at +2 V were recorded between CPT structures and surrounding PU regions. The pronounced 4° phase shift difference on Au/PU-g-CPT compared to the other surfaces highlights the strong electrostatic contribution of the CPT to bare gold-derived PU interface, as well as consistency with the aggregated morphology observed in AFM (Figure 3c). Although MUA/PU and MUA/PU-Acry exhibit fibrous topography, the EFM phase-bias analysis (Figure S11) shows that CPT-grafted surfaces display larger phase-shift magnitudes under applied bias than the corresponding PU-only controls. This behavior is consistent with a distinct electrostatic response after CPT incorporation. Together with the FTIR (Figure 5) and XPS S 2p data (Figure S8), these results support the assignment of the observed nanowire-like morphologies to CPT-containing surface structures. In addition, across all samples, 0 V reference EFM images exhibit negligible phase shifts, confirming the measurement reliability and eliminating topography-driven artifacts.

**Figure 5:**
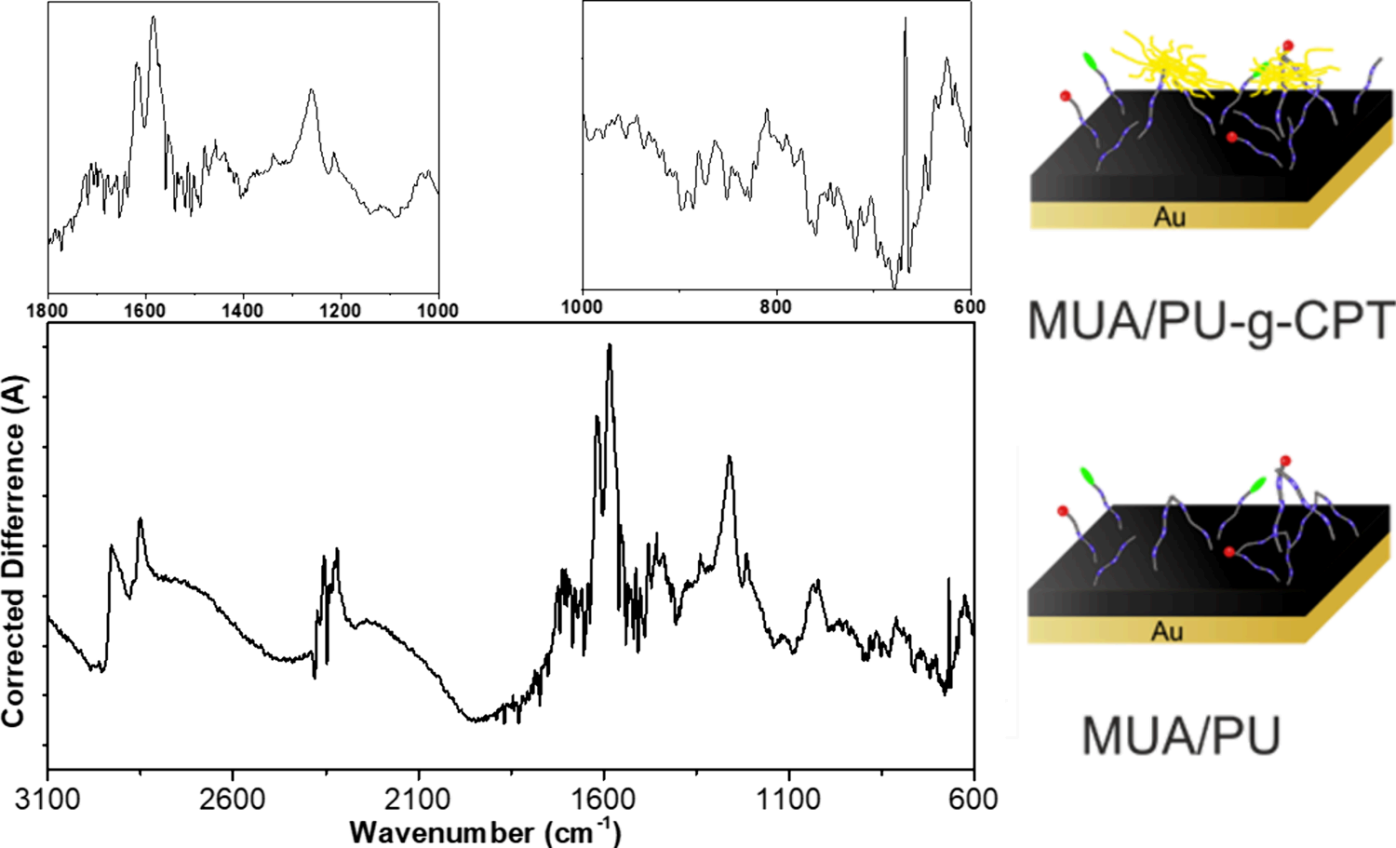
Subtracted FTIR spectrum (MUA/PU-g-CPT - MUA/PU) with corresponding schematic illustrations.

Functional group analysis of CPT was performed using VeeMAX III (variable angle specular reflectance accessory) combined with Fourier transform infrared (FTIR) spectroscopy. FTIR spectra of MUA/PU-g-CPT and MUA/PU were measured to confirm vibrational bands of CPT grafted on the gold surface. To demonstrate the CPT-specific peaks, a difference spectrum was obtained by subtracting FTIR spectra (MUA/PU-g-CPT – MUA/PU), as shown in Figure 5 (the original spectra of the surfaces are provided in Figure S12). The subtracted spectrum reveals in-plane C=C vibration of thiophene rings at 1403 cm^–1^ and 1225 cm^–1^. Symmetric C–S stretching appeared at 835 cm^–1^ while asymmetric C–S stretching was identified at 895 cm^–1^. An additional band at 760 cm^–1^ could be attributed to the ring deformation vibration of the thiophene units.^32^ These spectroscopic features corroborate the successful immobilization of CPT on the gold surface, in agreement with AFM and EFM analyses.

Figure 6 and Table 4 summarize the structural and chemical characteristics of the surfaces. Comprehensive characterization across four surfaces revealed a clear relationship between surface chemistry and the resulting nanowire architecture. Controlled PU growth on MUA-functionalized gold substrate, followed by UV-induced allyl-methacrylate coupling, enabled the formation of stable and highly elongated CPT nanowires with well-defined nanoscale morphology (Figure 6). XPS analysis showed that a 6-cycle SurfAst polymerization protocol yields the maximum PU coverage on MUA-functionalized gold, providing a chemically uniform and reactive interface for subsequent CPT grafting. AFM cross-section and surface profile analyses, which are summarized in Table 4, highlight the essential role of MUA in orienting the reactive groups at the interface and promoting nanowire growth. On MUA-functionalized PU interface (MUA/PU-g-CPT), CPT forms homogeneous structures characterized by central domains averaging 61 nm in height and elongated wire-like extensions tapering from these centers. In contrast, grafting directly onto the bare gold-derived PU interface (Au/PU-g-CPT) leads to disordered, aggregated features with diminished structural definition. These comparisons demonstrate that nanowire alignment, thickness, and overall architecture are influenced by substrate functionalization and SurfAst polymerization process. The solvent environment may act as an additional factor contributing to structural development. Solvation of CPT in ethylene glycol enhances polymer–solvent interactions, promotes molecular dispersion prior to grafting, suppresses aggregation, and thereby increases grafting density and structural homogeneity of the resulting nanowires. Electrostatic force microscopy provided complementary evidence of successful CPT grafting. CPT-bearing surfaces exhibited pronounced phase contrast at ±2 V tip bias, consistent with the cationic nature of CPT chains. FTIR spectroscopy further confirmed the presence of thiophene vibrational modes on the grafted surfaces, validating the chemical integrity of the CPT nanowires.

**Figure 6:**
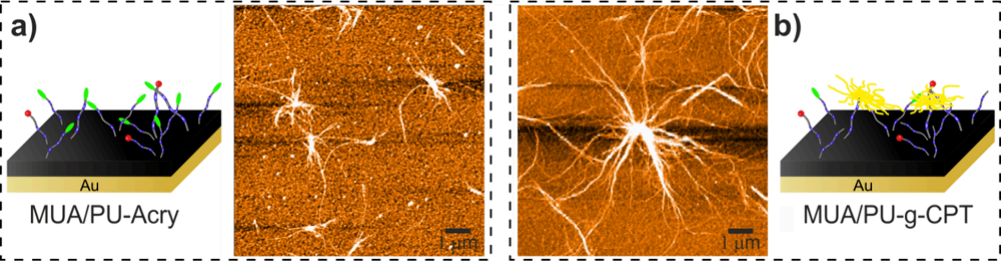
AFM topography images of (a) SurfAst-based PU interface (MUA/PU-Acry) and (b) CPT grafting on MUA/PU-Acry (MUA/PU-g-CPT) with corresponding schematic illustrations.

**Table 4:** Overall Comparison of AFM and EFM Parameters of the Surfaces

	MUA/PU-Acry	MUA/PU-g-CPT	MUA/PU	Au/PU-g-CPT
roughness (nm) (grafted area)	49.8 (±15)	93.3 (±35)	138 (±70)	123 (±43)
roughness (nm) (non-grafted area)	4.42 (±0.9)	3.35 (±0.9)	4.7 (±0.7)	23 (±4)
height center (nm)	38 (±8)	61 (±29)	53 (±14)	125 (±33)
			220 (±36)	
height fiber (nm)	10 (±2)	19 (±5)	10 (±3)	64 (±20)
		6 (±2)	11 (±2)	
width fiber (nm)	159 (±35)	265 (±110)	166 (±63)	290 (±33)
		58 (±40)	189 (±100)	
length fiber (μm)	2.0 (±0.5)	3.6 (±0.7)	1.6 (±0.2)	0.6 (±0.1)
			2.5 (±0.5)	
phase shift, –2 V (deg)	2.3 (±0.2)	3.0 (±0.3)	1.0 (±0.2)	5.6 (±0.4)
phase shift, +2 V (deg)	1.4 (±0.2)	2.3 (±0.3)	1.8 (±0.2)	1.6 (±0.2)

## Conclusion

In this study, we developed a robust and versatile strategy for fabricating cationic conjugated polythiophene (CPT) nanowires covalently grafted onto gold surfaces through surface-assisted (SurfAst) polymerization. Overall, these results present SurfAst polymerization as a powerful, modular approach for fabricating covalently grafted cationic polythiophene nanowires with various morphologies and electrostatic characteristics, showing a persistent CPT layer after extensive aqueous washing.. Beyond demonstrating precise nanoscale control, this work offers fundamental insight into conjugated polyelectrolyte-surface interactions and provides an approach for integrating CPT nanostructures into advanced electronic, sensing, and biointerface technologies.

## Supplementary Material



## Notes

Details of the experimental section, additional AFM topography images, cross-section analysis (Gwyddion-reprocessed AFM images for comparison), XPS chemical mapping, AFM topography image of CPT, SANS curves, high-resolution S 2p XPS and XPS survey spectra phase-tip voltage graph, phase shift-tip bias graph, and original FTIR spectra of the surfaces, MUA/PU-g-CPT and MUA/PU (PDF)

## ‡

Institute for Complex Molecular Systems (ICMS), Department of Chemical Engineering and Chemistry, Eindhoven University of Technology, 5600 MB Eindhoven, Netherlands
