# Supplementary material for: Covalent Grafting of Cationic Polythiophene Nanowires onto a Polyurethane Interface via Surface-Assisted Polymerization
Source: Langmuir. 2026 Jun 12;42(25):17809–18. doi: 10.1021/acs.langmuir.6c00484 (PMC13325862; doi:10.1021/acs.langmuir.6c00484)
Supplement: Supplementary file 1 [file la6c00484_si_001.pdf]

[Supporting Information]

Covalent Grafting of Cationic Polythiophene Nanowires onto Polyurethane Interface via Surface-Assisted Polymerization

Sezer Özenler[a], Muge Yucel[b], Ümit Hakan Yıldız[a,c,d]**

a. Department of Chemistry, Izmir Institute of Technology, Urla, 35430, Izmir, Turkey

b. Department of Bioengineering, Izmir Institute of Technology, Urla, 35430, Izmir, Turkey

c. Department of Photonics, Izmir Institute of Technology, Urla, 35430, Izmir, Turkey

d. Polymer Science and Engineering Program, Izmir Institute of Technology, Urla, 35430, Izmir, Turkey

KEYWORDS: Cationic Polythiophenes, Nanowires, Surface-Assisted Polymerization, Graft-to, Gold Surface

Materials and Methods: Poly(N-allyl-N-methyl-N-(3-((4-methylthiophen-3-yl)oxy)propyl)prop-2-en-1-aminium bromide as cationic polythiophene was synthesized as described elsewhere.[1] **Dibutyltin dilaurate (DBTDL) catalyst:** 11.2 mg DBTDL is dissolved in 15.4 ml acetone as a stock solution. All gold substrates are cleaned by the RCA cleanser as previously described.[3]

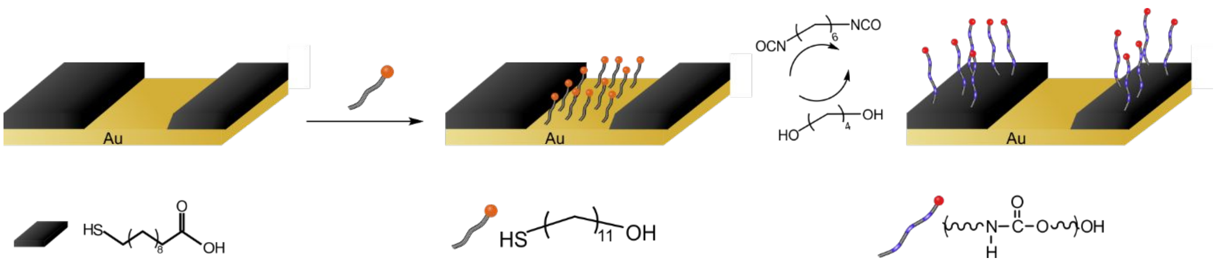

Scheme S1. Schematic illustration of stamped-Au/MUA/PU-OH fabrication.

Stamped-Au/MUA/PU-OH: The methods for stamping are carried out as previously described.[2] 11-mercaptoundecanoic acid (MUA) is utilized to functionalize the gold surface by stamping with square-shaped polydimethylsiloxane (PDMS). 1 mM 11-mercapto-1-undecanol is used for backfilling in the ethanolic solution (EtOH) for an overnight period on MUA-functionalized gold surface and plenty of EtOH is used to rinse the gold surface. The first procedure involves incubating the gold surface with HDI (70 µl in 5 ml acetone, 200 µl DBTDL catalyst) in acetone at 40˚C for 20 minutes. The second stage involves incubating 1,4-BDO (40 µl in 5 ml acetone, 200µl DBTDL catalyst) in acetone at 40˚C for 20 minutes on the gold surface. The first and second stages are repeated four times, respectively (Scheme S1).

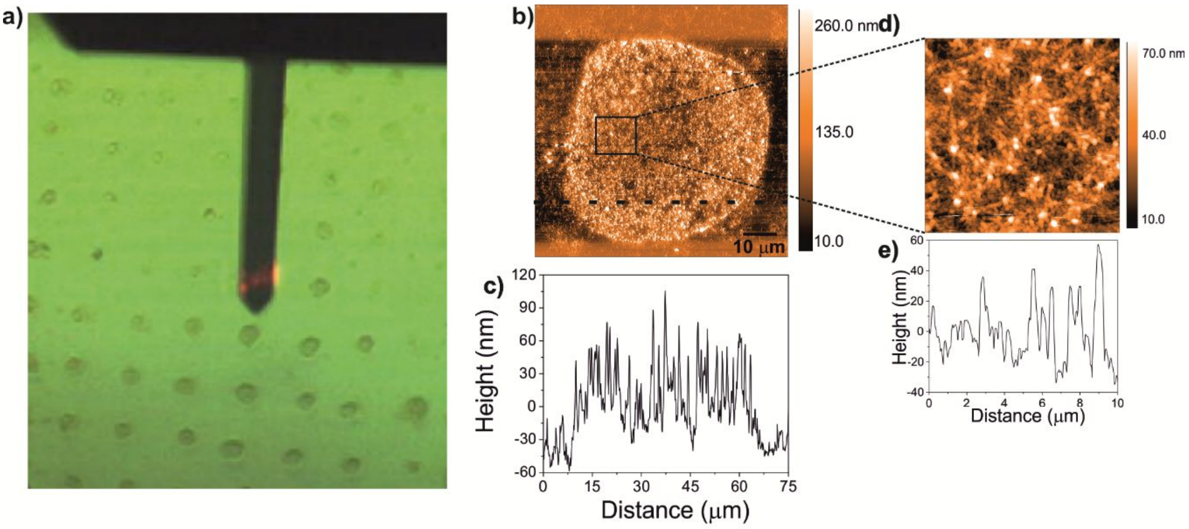

Figure S1. (a) Microscope image, (b,c) AFM topography image and cross-section (black dashed line) (d,e) AFM height image and cross-section (black dashed line) of a magnified view of stamped-Au/MUA/PU-OH (Square-shape MUA-functionalized gold surface was passivated with 11-mercapto-1-undecanol).

Figure S1 shows the AFM topography and microscope image of four sequential reactions between 1,4-BDO and HDI on the gold surface. After sequential incubations yield nanoporous PU interface on MUA-functionalized gold surface as shown in figure S1b and d. Cross-section image shows the height distribution is around 130 nm in Figure S1c. In Figure S1d, the PU interface demonstrates bundles like structure gathering polymer chains. The height values of the PU interface are observed to vary between 30 and 90 nm. The average surface roughness is 150 nm. Results show that a bundle-like PU structure is obtained by applying the SurfAst urethane polymerization to the MUA-functionalized gold surface.

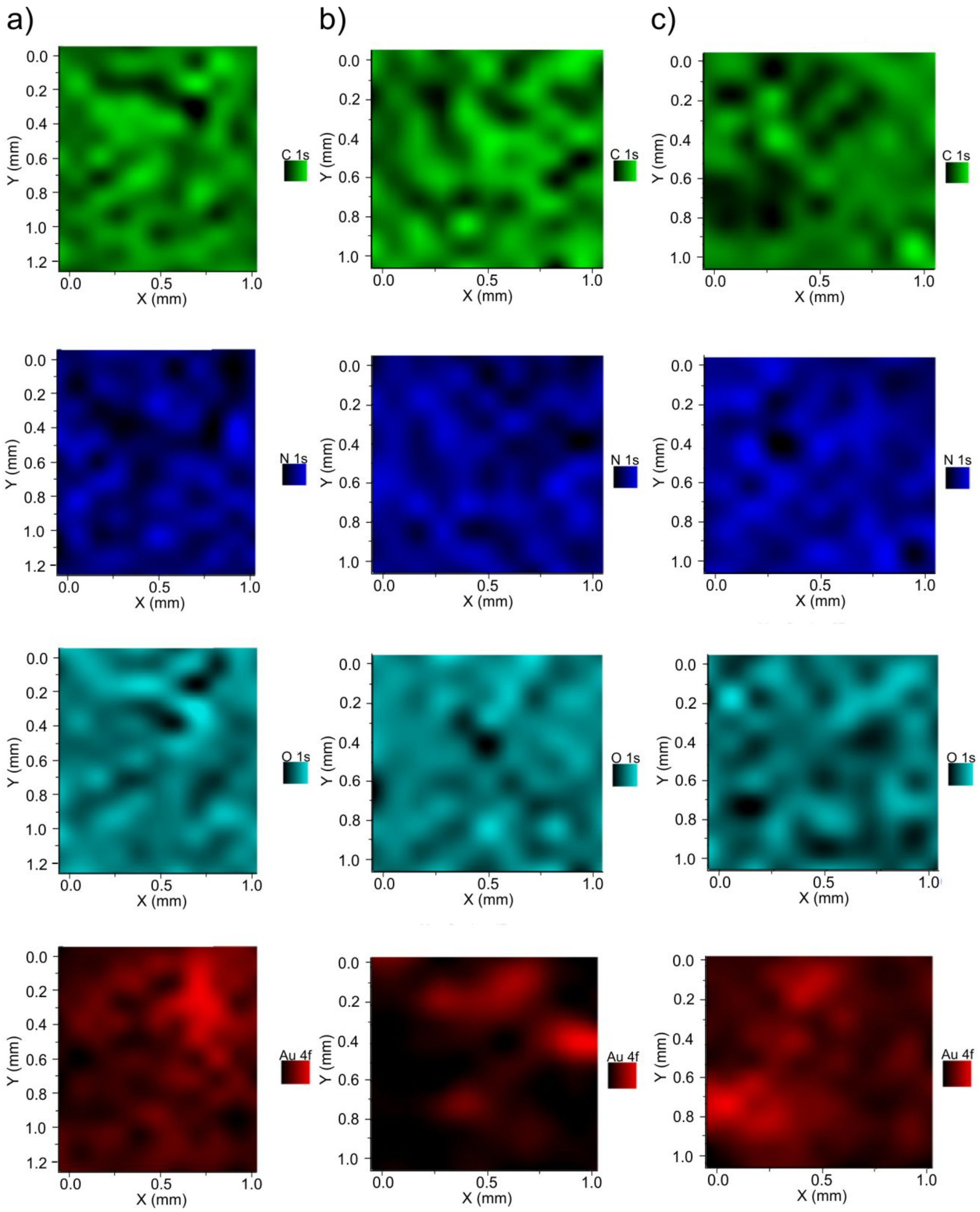

Figure S2. XPS chemical mapping of bare gold surface after a) 4, b) 6, and c) 8 cycle HDI/1,4-BDO incubation. Color coding: green; C1s, blue; N 1s, cyan; O 1s, and red; Au 4f.

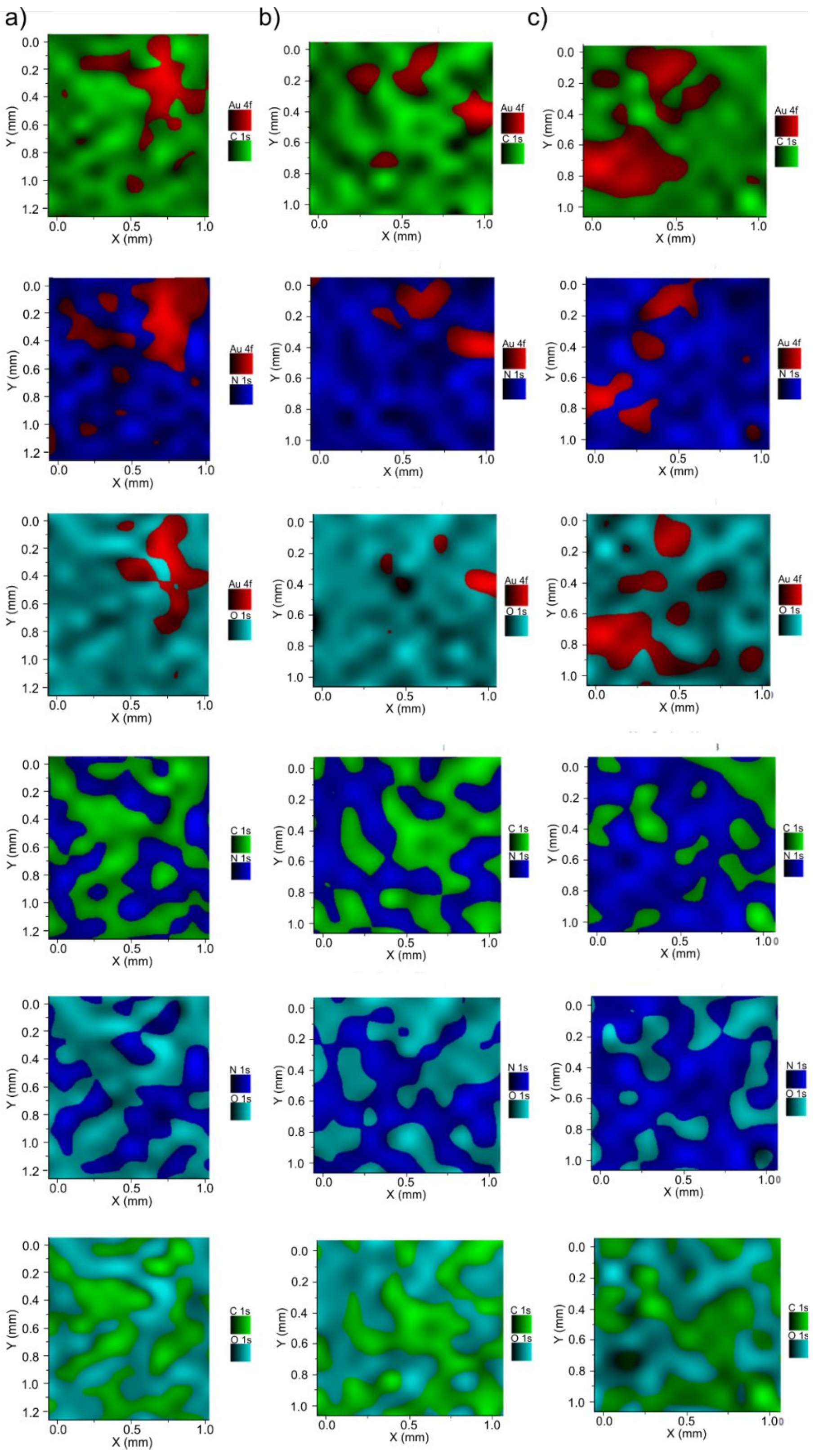

Figure S3. XPS binary chemical mapping of bare gold surface after a) 4, b) 6, and c) 8 cycle HDI/1,4-BDO incubation. Color coding: green; C1s, blue; N 1s, cyan; O 1s, and red; Au 4f.

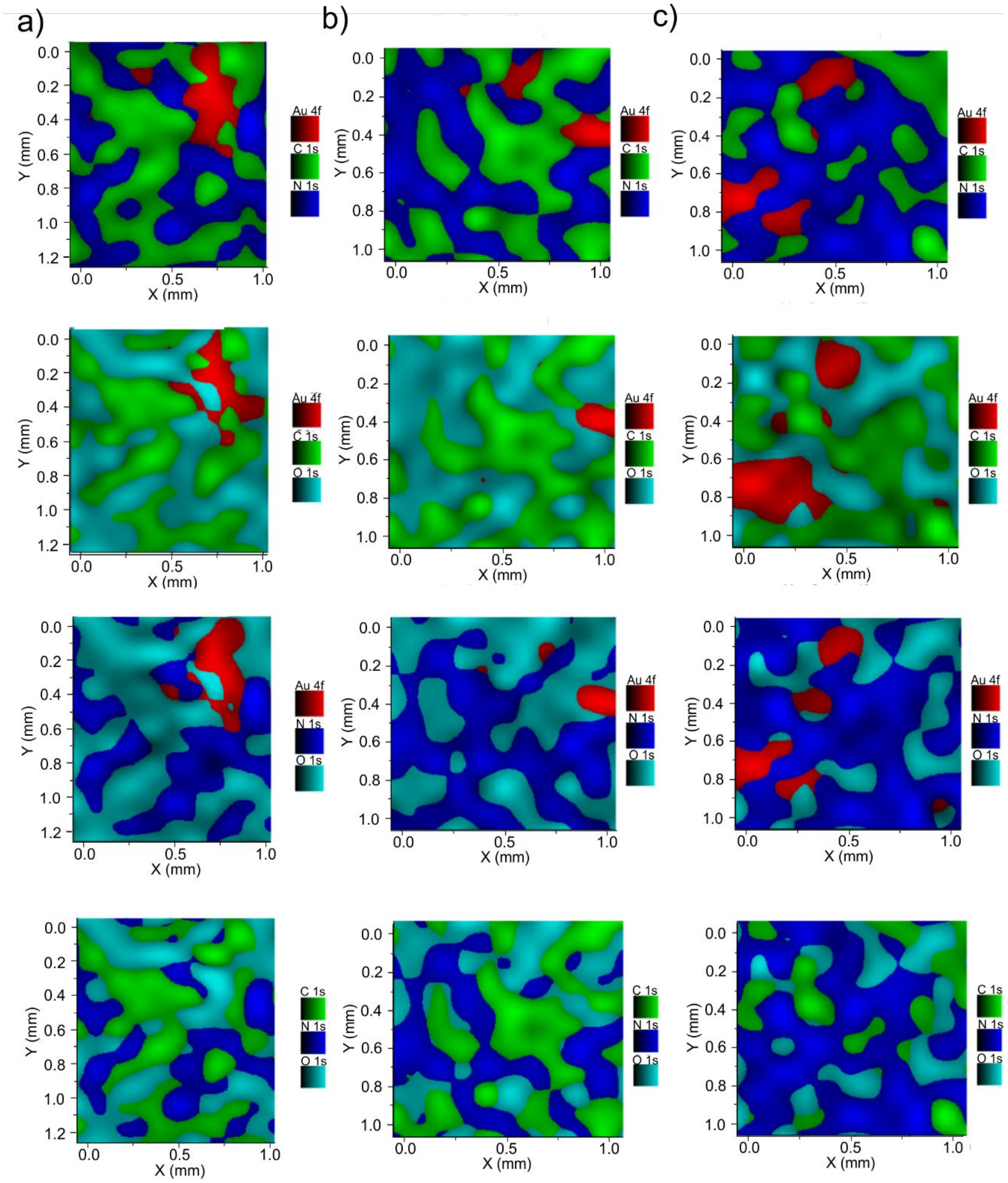

Figure S4. XPS tertiary chemical mapping of bare gold surface after a) 4, b) 6, and c) 8 cycle HDI/1,4-BDO incubation. Color coding: green; C1s, blue; N 1s, cyan; O 1s, and red; Au 4f.

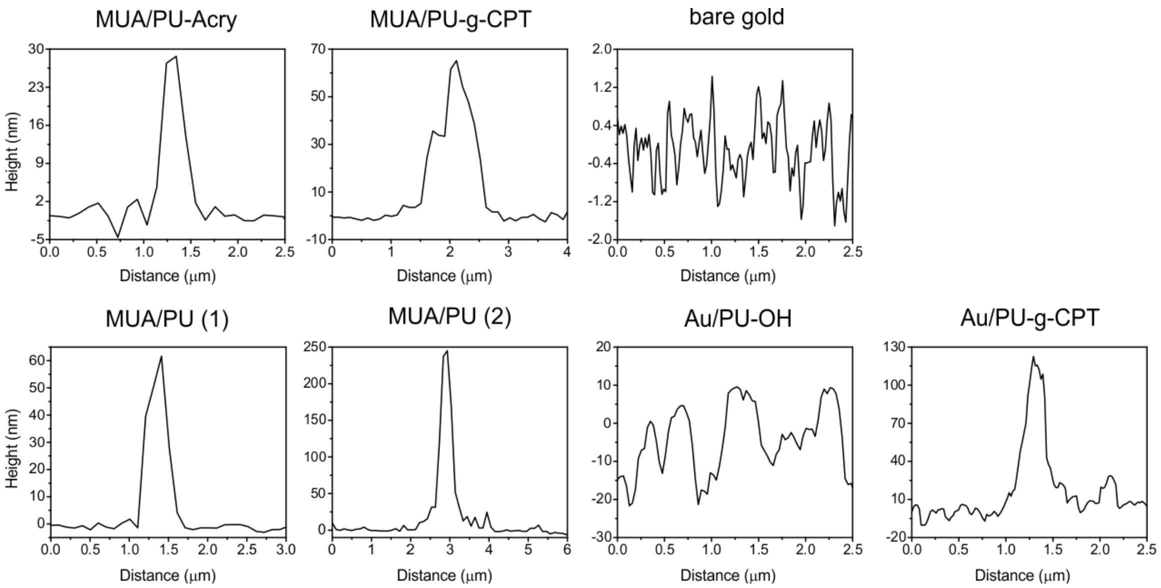

Figure S5. Cross-section analysis of surfaces and bare gold (the lines where the analysis performed are shown in Figure 2 and 3 as cyan dashed lines.

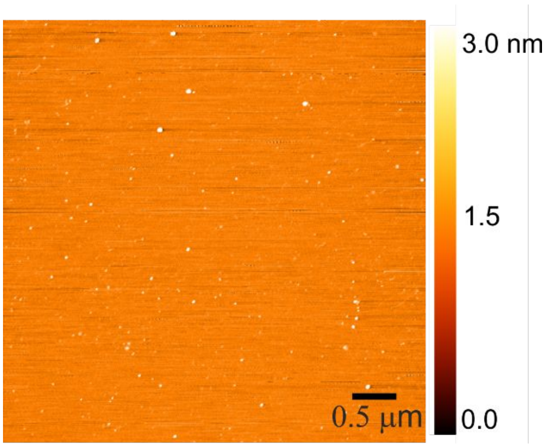

Figure S6. Figure S6. AFM topography image of CPT deposited from ethylene glycol solution onto a gold surface.

Small Angle Neutron Scattering: SANS measurements were performed using the D22 instrument (https://doi.ill.fr/10.5291/ILL-DATA.9-12-699) at the Institut Laue Langevin (ILL) in Grenoble, France. CPTs were dissolved in D_2O and EG-d6 solutions to yield 2 mg/ml polymer solution. For the SANS measurement, the instrument parameter was set up as following: neutron wavelength λ = 6.0 Å, sample–detector distances (1.4 m, 17.6 m) with

collimation distance of 17.6 m. The samples were measured for 90 mins in a scattering vector range of 0.026 $Å^{-1} \leq q \leq 0.64$ $Å^{-1}$.

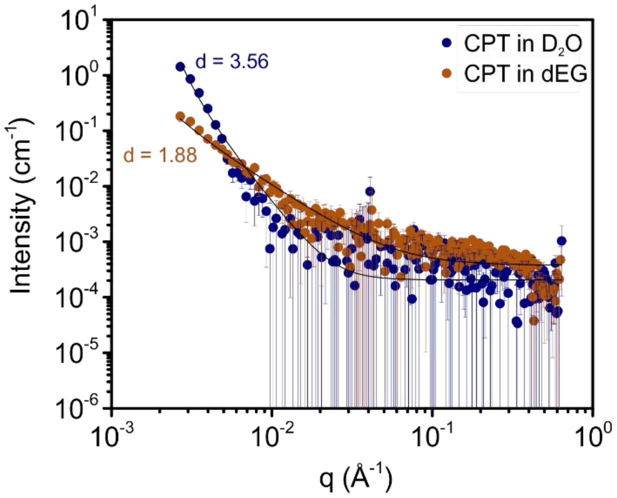

Figure S7. SANS curves of conjugated polythiophene (CPT) dissolved in d6-EG and D_2O with power-law exponentials calculated by the SasView.

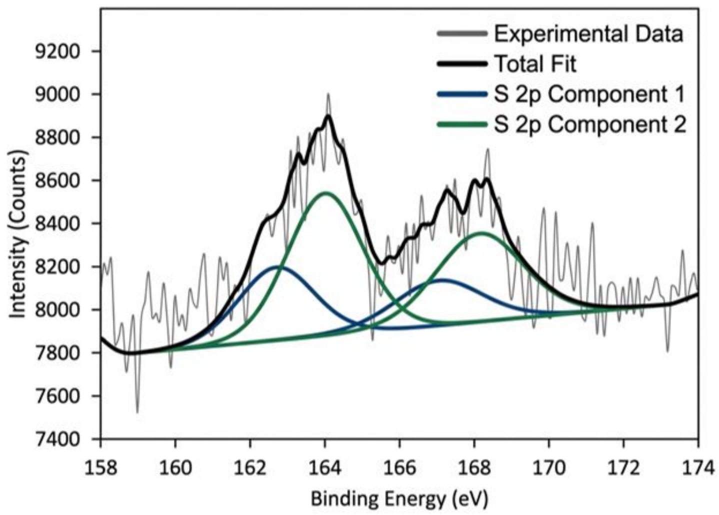

Figure S8. High-resolution S 2p XPS spectrum of the CPT-grafted surface.

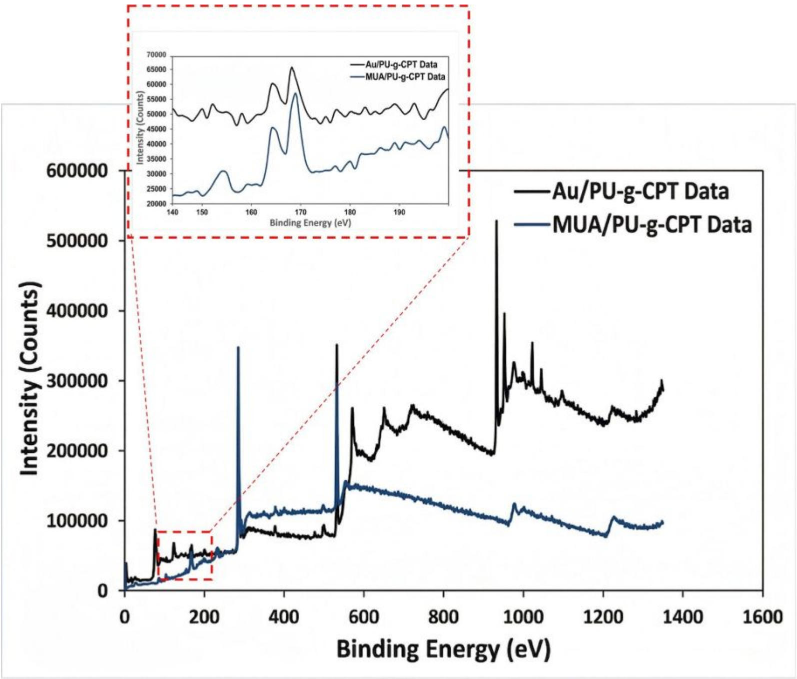

Figure S9. XPS survey spectra of Au/PU-g-CPT and MUA/PU-g-CPT after two weeks of ambient exposure.

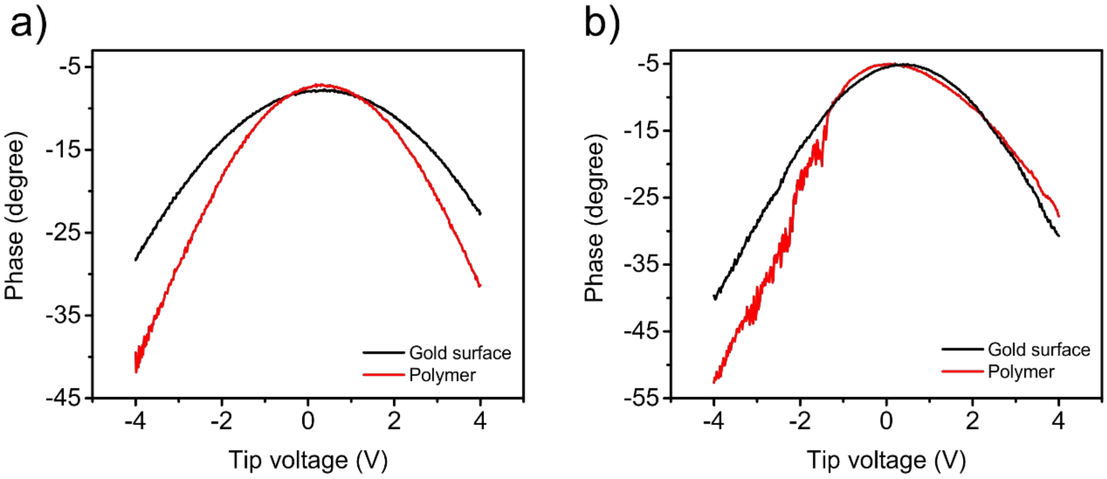

Figure S10. Phase-tip voltage graph of (a) MUA/PU-Acry and (b) MUA/PU.

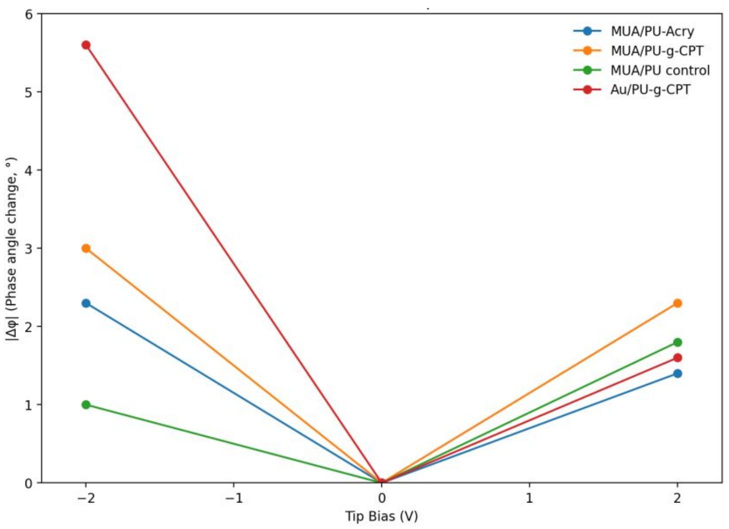

Figure S11. Absolute phase-shift magnitude (|Δφ|) as a function of applied tip bias for surfaces.

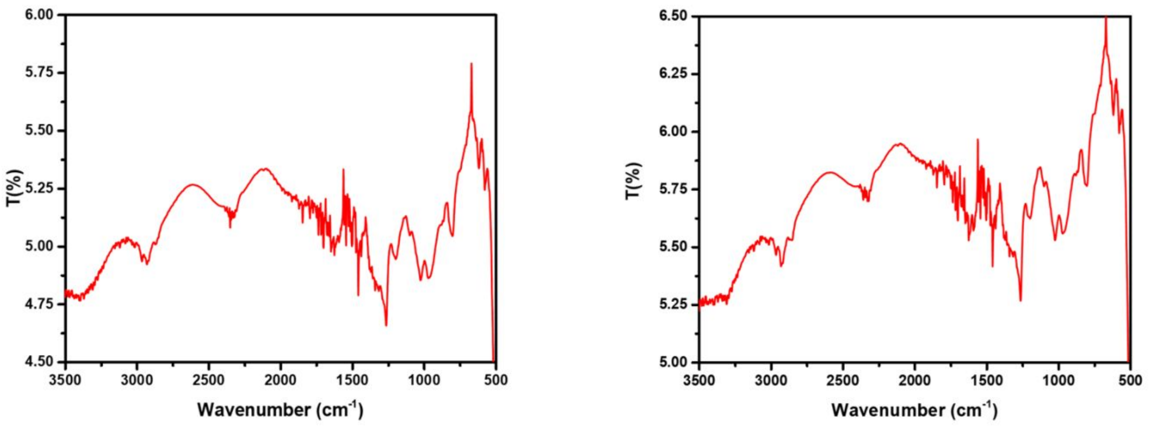

Figure S12. The FTIR spectra of the surfaces, (left) MUA/PU-g-CPT and (right) MUA/PU.

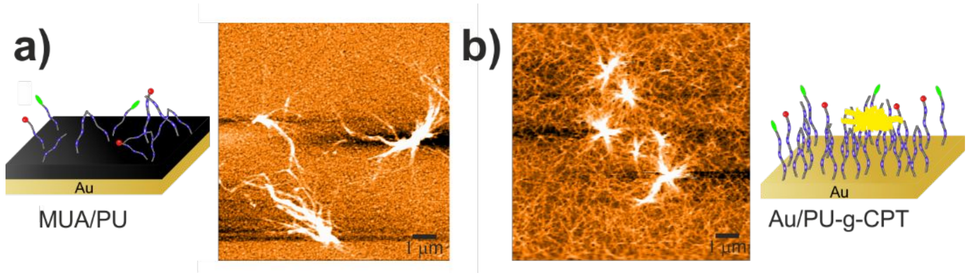

Figure S13. AFM topography images of a) **MUA/PU** (control surface of MUA/PU-g-CPT) and b) **Au/PU-g-CPT** with corresponding schematic illustrations.

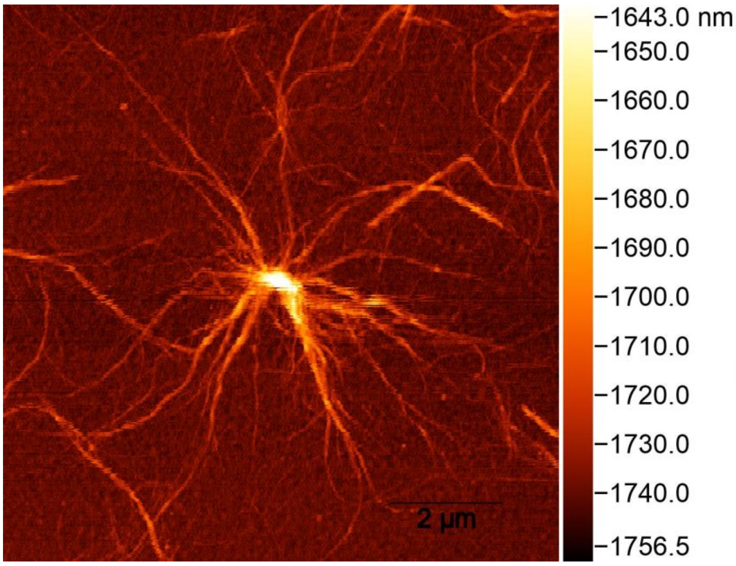

Figure S14. AFM topography image of CPT grafting on **MUA/PU-Acry** (**MUA/PU-g-CPT**), reprocessed using masked flattening in Gwyddion.

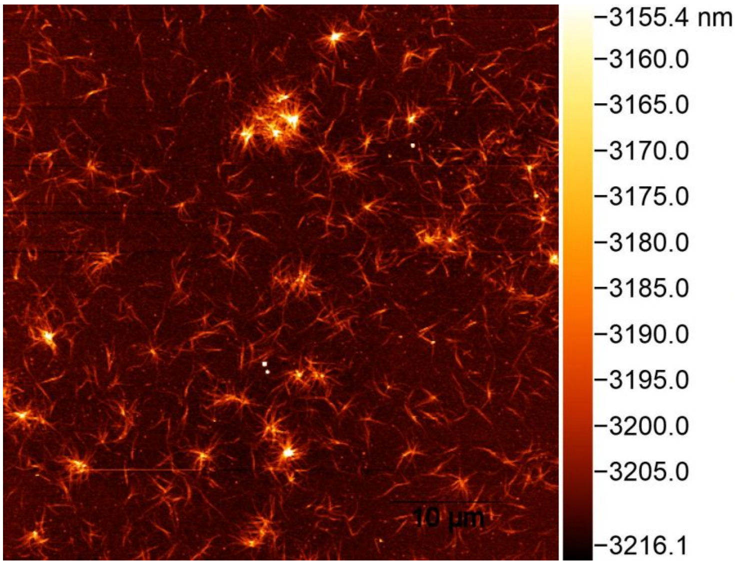

Figure S15. AFM topography image of **MUA/PU-g-CPT**, reprocessed using masked flattening in Gwyddion.

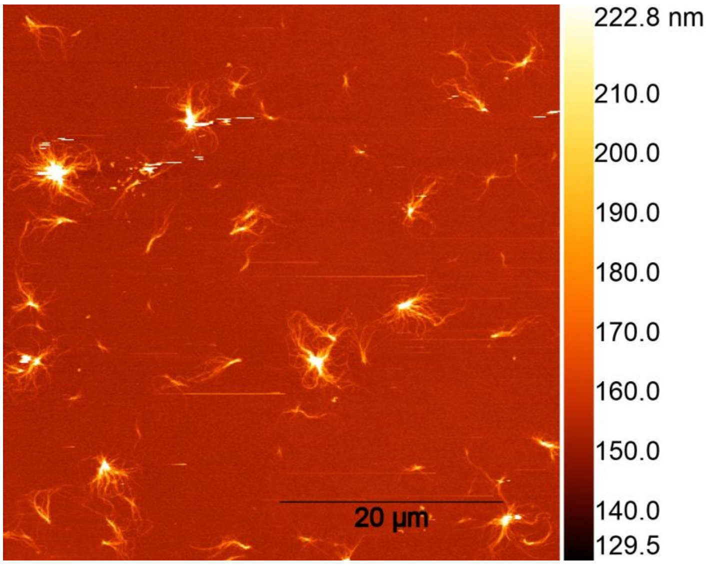

Figure S16. AFM topography image of **MUA/PU**, reprocessed using masked flattening in Gwyddion.

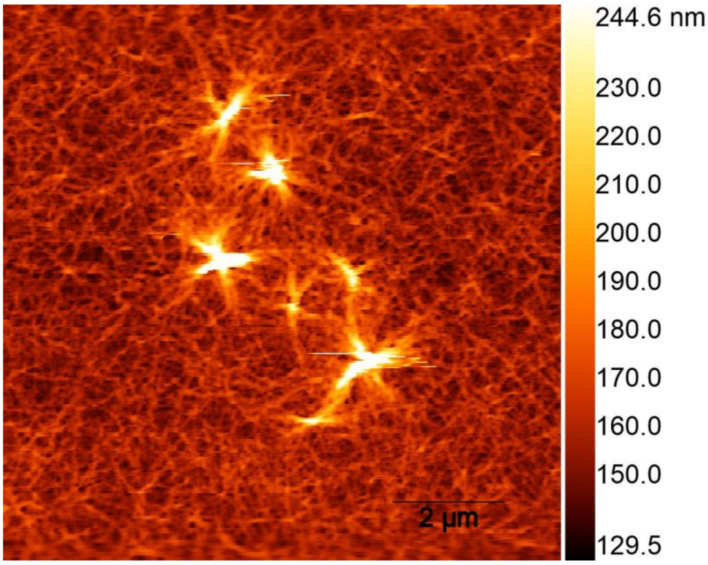

Figure S17. AFM topography image of **Au/PU-g-CPT**, reprocessed using masked flattening in Gwyddion.

References

1. Yucel, M., Koc, A., Ulgenalp, A., Akkoc, G. D., Ceyhan, M., Yildiz, U. H. (2021). PCR-free methodology for detection of single-nucleotide polymorphism with a cationic polythiophene reporter. ACS sensors, 6(3), 950-957.

2. Ozenler, S., Sozen, Y., Sahin, H., Yildiz, U. H. Fabrication of a postfunctionalizable, biorepellent, electroactive polyurethane interface on a gold surface by surface-assisted polymerization. Langmuir 2020, 36(24), 6828-6836.
